# The role of self-assessment in the formation of communication style in middle school children

**DOI:** 10.3389/fpsyg.2026.1846073

**Published:** 2026-08-12

**Authors:** Saule Alimbayeva, Klara Smatova, Zhanar Sabraliyeva, Gaukhar Sanay, Altynbek Boltayev, Elmira Aitenova

**Affiliations:** 1 Taraz Regional University named after M.KH.Dulaty, Taraz, Kazakhstan; 2 Department of Pedagogical Psychology, Faculty of Pedagogy and Social Sciences, Taraz Regional University named after M.KH.Dulaty, Taraz, Kazakhstan; 3 Department of Special and Social Pedagogy, Faculty of Pedagogy and Social Sciences, Taraz Regional University named after M.KH.Dulaty, Taraz, Kazakhstan; 4 Department of Educational Psychology, Faculty of Pedagogy and Social Sciences, Taraz Regional University named after M.KH.Dulaty, Taraz, Kazakhstan

**Keywords:** adolescence, assertiveness, communication style, gender differences, psychological wellbeing, regional factors, self-esteem

## Abstract

**Background:**

Adolescence (ages 14–15) is a critical period for the formation of self-concept and communicative competence. While previous research has explored these constructs independently, the relationship between self-esteem and communication styles in the context of gender and regional factors remains insufficiently studied, particularly within standardized educational frameworks.

**Methods:**

This study utilized the ‘I-psychodiagnostics.kz’ SMART platform to assess 303 students (*N* = 303) using the Piers–Harris Children’s Self-Concept Scale and the Children’s Assertive Behavior Scale (CABS). Data were analyzed using descriptive statistics, Student’s *t*-tests, and one-way ANOVA.

**Results:**

Self-perception among girls and boys was assessed using the Piers–Harris Children’s Self-Concept Scale. The girls demonstrated more positive self-evaluations in the Intellectual and School Status domain (*M* = 12.69). In contrast, the boys scored higher in Behavioral Adjustment (*M* = 11.64). These findings confirm the absence of statistically significant gender differences, as adolescent development is more heavily shaped by the immediate social circle, the need for peer acceptance, and the institutional school environment. The assessment of tendencies toward non-assertive behavior also revealed no significant differences between the cohorts. Such tendencies were found to be context-dependent. Furthermore, self-esteem levels influenced communication styles regardless of gender. High self-esteem had the most substantial impact on the active-constructive communication style, as observed in both girls (91%) and boys (94%). High self-esteem was also associated with advanced communication proficiency. This relationship remained independent of regional factors (students from schools located in different settlements) or the status of the educational institutions (*F* = 1.01).

**Conclusion:**

The results suggest that internal psychological resources, specifically self-esteem, are more critical for developing communicative confidence than external environmental or institutional factors. These findings provide a robust empirical basis for developing school-based interventions focused on enhancing self-regulatory capacities and socio-emotional learning.

## 1 Introduction

The social landscape of modern society is profoundly influenced by factors that shape and transform an individual’s communication style. Social interaction, including engagement via social media, is a cornerstone of identity development (Sebre and Miltuze, 2021; Senekal et al., 2023) and personality formation, contributing significantly to adolescent character acquisition. Communication style serves as the foundation of interpersonal interaction; it plays a vital role in shaping worldviews and determines the trajectory of social skills development. These skills include self-confidence, tolerance of criticism, social adaptability, flexibility, emotional regulation, and overall interpersonal efficacy. Key personality traits, such as extraversion and conscientiousness, play a multifaceted role in this process. They influence communication styles by contributing to expressiveness and accuracy (Dhillon and Kaur, 2023). Conversely, these traits can transform under the influence of the specific social interaction tactics an individual adopts. Self-esteem is interpreted as one of the most influential aspects governing social development (Poudel et al., 2020) and is considered the ‘source of social behavior’ (Rosenberg et al., 1989). Furthermore, self-esteem plays a key role in psychological well-being and life satisfaction (Szcześniak et al., 2022), which largely determines an individual’s degree of self-disclosure, demeanor, and communication style.

During adolescence, communication styles are intrinsically linked to students’ self-confidence and psychological development (Karadaş et al., 2026). This developmental stage is often characterized by the emergence of interpersonal challenges, such as conflict, social withdrawal, or bullying. The adjustment of self-esteem levels serves as a mechanism to refine adolescent communication styles by enhancing self-perception. Furthermore, healthy self-esteem fosters greater openness and a readiness for constructive engagement. Effective communication skills significantly influence peer acceptance within the classroom and enhance academic motivation by fostering psychological well-being (Zhubakova et al., 2025). Ultimately, high self-esteem contributes to the mitigation of conflicts and socialization difficulties.

According to sociometer theory, self-esteem can be viewed as a psychological parameter of an individual’s social acceptance. This is associated with individuals’ evaluation of their perceived position within their social environment. The development of self-evaluation among school students is associated with their experience of communication with other students and influences the constructiveness of this process (Perinelli et al., 2022). Sociometer theory is also supported by George Herbert Mead’s theory of the social self, which suggests that an individual’s self-concept depends on the level of social interaction. This occurs through receiving responses from others and forming perceptions of one’s social efficacy, which influences the characteristics of communication with other people (Côté, 2023).

Given the rapid pace of digitalization, a substantial portion of communication has transitioned to virtual spaces. This shift results in a reduction of face-to-face peer interaction. Consequently, this lack of direct contact manifests as changes in the cohesion of interpersonal relationships. The effectiveness of social interaction in virtual environments is highly dependent on the level of ‘digital maturity’ (Koch et al., 2025). In contrast, within the context of offline, face-to-face communication, self-esteem potentially plays a more pivotal role.

Specific psychological mechanisms underlie the linkage between these two constructs. Self-esteem shapes how adolescents perceive their own social competence and confidence during interactions with others. Adolescents with a more positive self-concept tend to initiate communication more frequently, express their views openly, and adopt more constructive interaction strategies (Tang et al., 2025). Diminished self-esteem, by contrast, frequently co-occurs with insecurity, heightened sensitivity to social evaluation, and the adoption of less effective communicative strategies. This psychological construct additionally links to the dynamics of social adaptation and the processing of peer feedback (Noman et al., 2025). Given that adolescence involves a heightened emphasis on interpersonal acceptance, positive social experiences can reinforce self-esteem, whereas negative encounters contribute to communication difficulties (Perinelli et al., 2022). Self-esteem and communication styles may thus be viewed as interrelated psychological characteristics reflecting an adolescent’s adaptation to the social environment.

Sociometer theory explains this linkage by positing that self-esteem reflects an individual’s subjective perception of personal social worth. Self-esteem is dynamic rather than static during its developmental period. The presence of low (underestimated) or inflated (overestimated) self-esteem in a child requires targeted diagnostic attention for the timely correction of maladaptive communication patterns. However, as one of the most malleable psychological properties, self-esteem can serve as a catalyst for refining communication styles during early and middle adolescence. Transformations in communication habits entail changes in how individuals perceive their future societal roles and efficacy, which necessitates further empirical investigation and justifies the focus of this study.

A review of the theoretical framework reveals a significant research emphasis on the formation of self-esteem. However, the link between self-esteem and communication styles remains insufficiently explored. The lack of established theoretical mechanisms integrating these two domains is reflected in a scarcity of practice-oriented studies. This scientific gap underscores the necessity for a systematic investigation of adolescent behavior, specifically focusing on gender-based differences. The purpose of this study is to examine the role of self-esteem in the formation of communication styles among middle school-aged children. Additionally, it seeks to identify the correlations between these aspects, using the personal development experience of adolescents in the Republic of Kazakhstan as a case study. To investigate self-esteem and communication skills, we utilized the ‘I-psychodiagnostics.kz’ SMART-platform. The data collected were used to test the following hypotheses:

Н0: Middle school-aged children exhibit a weak dependence of communication style formation on self-esteem, suggesting its limited importance in the development of communicative skills (*M*_1_ = *M*_2_).

Н1: Middle school-aged children are characterized by a moderate-to-strong dependence of communication style formation on self-esteem (M_1_ ≠ M_2_).

### 1.1 Hypotheses regarding gender

H₀: There are no statistically significant differences in self-esteem and communication style scores between middle-school boys and girls (M_1_ = M_2_).

H₁: There are statistically significant differences in self-esteem and communication style scores between middle-school boys and girls (M_1_ ≠ M_2_).

### 1.2 Hypotheses regarding the regional factor

H₀: There are no statistically significant differences in self-esteem and communication scores between children attending different schools (M_1_ = M_2_).

H₁: There are statistically significant differences in self-esteem and communication scores between children attending different schools (M_1_ ≠ M_2_).

## 2 Literature review

According to the Children’s Assertive Behavior Scale (CABS), developed by Michelson and Wood (Michelson and Wood, 1982), communication styles can be categorized into three primary types: assertive, passive, and aggressive. An assertive communication style is fundamental to the development of robust communication skills. It facilitates a balanced range of reactions, which collectively enable individuals to act appropriately based on the requirements of various communicative situations. Deviations from assertiveness are assessed using the Aggressive Behavioral Assessment Scales (AGCABS) and Passive Behavioral Assessment Scales (PACABS). Higher scores on the general CABS scale indicate a greater degree of deviation from adaptive social interaction, manifesting as either aggression or passivity. de la Torre et al. (2021) stated that assertive behavior is shaped by an awareness of personal rights, the ability to defend them, and the use of skills that allow an individual to remain objective and respectful toward themselves and others, without resorting to manipulation, aggression, or maladaptive reactions. Assertiveness is a personality trait that enables authentic self-expression, specifically regarding feelings, ideas, and opinions (de la Torre et al., 2021), Together, these elements form an assertive communication style and contribute to the cohesion of interpersonal relationships. In addition to aggression and passivity, children may be prone to avoidance due to various factors, such as excessive demands or expectations (Pérez et al., 2021). However, existing research lacks sufficient experimental evidence linking self-esteem to communication styles among schoolchildren. Detachment is a more severe form of passivity, often manifesting as social anxiety or a method of avoiding interaction (Marici et al., 2024). During the formation of a communication style, a child may experience psychological transformations and negative emotional outcomes, including increased anxiety, shame, and a general decline in self-identity and perceived self-worth (Marici et al., 2023). External environmental influences shape both personality and self-esteem, which in turn affect social interaction and the choice of behavioral patterns. This suggests a bidirectional interdependence between these categories; therefore, determining the role of self-perception in shaping communication styles requires additional research into specific aspects of a child’s self-esteem.

Factors affecting communication style, behavior, socialization, and psycho-emotional health are widely investigated in contemporary psychology. A generalized classification provided by Smare and Elfatihi (2024) highlights four dimensions: individual, family, educational, and sociocultural. Individual aspects are particularly relevant to this study. Peng et al. (2022) described the significance of children’s dissatisfaction with their physical appearance, noting that self-criticism regarding body image is a primary driver of aggression and anger. Similarly, Merino et al. (2024) examined body dissatisfaction in the context of psychological health, highlighting deleterious outcomes such as depression and anxiety. Prince et al. (2024) identified low self-esteem related to physical appearance as a consequence of cyberbullying, leading to impairments in communication skills and overall well-being. However, these studies do not adequately address the relationship between self-esteem and specific proficiency levels of communication skills.

The role of intellectual and school status in behavior is described by Zajenkowski et al. (2022). The authors analyzed the relationship between intellectual development, plasticity, and extraversion, confirming these correlations in adults. Their results indicated that the development of self-esteem is dependent on cognitive development, which is further reflected in communication skills.

A study involving 533 students (Hina et al., 2025) demonstrated that the socialization process had a significant impact on self-esteem in 7.3% of the cases. This was particularly evident among students who actively participated in school events, sports, and extracurricular activities. However, a notable limitation of that study was its failure to address the influence of self-esteem on adolescent communication styles.

Furthermore, research (Yahi, 2025) suggests that low self-esteem is correlated with aggressive behavior in schoolchildren. During adolescence, such behavior often serves as a defense mechanism, providing a compensatory sense of confidence. A comparison between genders revealed higher aggression levels among boys, which was attributed to a need for tension release and underlying issues in emotional regulation.

A critical analysis of existing literature highlights a predominant theoretical focus on self-esteem in schoolchildren. Potential research gaps stem from a lack of focus on how self-esteem levels influence the dynamics of interaction among peers and between students and teachers, especially when considering regional and gender-based factors. Current publications lean toward theoretical frameworks of self-esteem, leaving a scarcity of empirical evidence regarding its direct link to communicative behavior.

## 3 Methodology

### 3.1 Methods

Achieving the research objectives relied on a comprehensive methodological approach that integrates theoretical methods (literature content analysis, generalization, and synthesis) and quantitative empirical methods. Statistical processing of the data included correlation analysis, Analysis of Variance (ANOVA), Student’s *t*-tests and *χ*^2^. The empirical component involved a structured survey of middle school-aged children conducted via the ‘I-psychodiagnostics.kz’ SMART platform.

### 3.2 Design

The study was conducted in two distinct phases:

*Phase* I: Theoretical Framework. This stage involved a comprehensive review of academic literature addressing the role of individual psychological characteristics in shaping communication styles. Particular emphasis was placed on the function of self-esteem within this framework. Existing research on communication styles and the psychological factors influencing their development was synthesized. This phase allowed for the formulation of preliminary constructs regarding the role of self-esteem in developing communicative skills and identified existing research gaps, specifically the degree of interdependence between self-esteem and communication styles during the psycho-emotional transformations of middle adolescence. Theoretical analysis was conducted during the preparation of the Introduction and Literature Review sections, which facilitated the evaluation of existing approaches to assessing school students’ self-esteem, the identification of research gaps, and the formulation of hypotheses for the subsequent empirical study.

*Phase* II: Empirical Investigation. The empirical study utilized the ‘I-psychodiagnostics.kz’ SMART platform to conduct large-scale testing. This platform was selected for its operational efficiency, user-friendly interface, and ability to provide structured data interpretation, which is essential for managing a significant sample size. Two standardized instruments were employed: the Piers–Harris Children’s Self-Concept Scale and the Children’s Assertive Behavior Scale (CABS).

To ensure analytical rigor, the evaluation of the relationship between self-esteem and communication styles (categorized by gender and regional factors) involved a comparative analysis (Tables 1–4). Data collection and the determination of communication styles were facilitated through direct collaboration with 11 educational psychologists. These experts assessed students’ self-esteem and communicative conduct using a triangulation approach: results from the standardized testing were supplemented by 45-min interviews and long-term observation of students’ school behavior prior to the formal investigation.

**Table 1:** Descriptive analysis of respondents’ responses on the Piers–Harris Self-Concept Scale by gender (*N* = 303).

Peirce-Harris Self-Concept Scale subscales	Answers, girls		Answers, girls	
	Mean	SD	Mean	SD
Physical appearance assessment (X_1_)	8.31	1.77	8.96	1.79
Intellectual status assessment (X_2_)	12.69	2.09	10.03	2.11
Happiness assessment (X_3_)	5.05	1.44	5.98	1.58
Emotional status assessment (X_4_)	9.06	1.95	10.19	1.82
Behavioural assessment (X_5_)	10.63	1.78	11.64	1.96
Popularity assessment (X_6_)	8.67	1.68	9.96	1.69
Total assessment	54.41	4.59	56.76	4.21

**Table 2:** Results of one-factor variance analysis.

Results				
Groups	Score	Total	Mean	Dispersion
Girls’ evaluation	6	54.41	9.0683	6.4872
Boys’ evaluation	6	56.76	9.4600	3.6441

Variance analysis						
Variation source	SS	df	MS	F	*p*-value	*F* critical
Between the groups	0.460208	1	0.4602	0.0908	0.7693	4.9646
Within the groups	50.65668	10	5.0657			
Total	51.11689	11				

**Table 3:** Descriptive analysis of respondents’ responses on the Assertive Behavior Scale (CABS) by gender (*N* = 303).

Evaluation criteria	Answers, girls		Answers, boys	
	Mean	SD	Mean	SD
Passive behavioural evaluation (PACABS; X_7_)	10.78	5.66	9.27	6.12
Aggressive behavioural evaluation (AGCABS; X_8_)	5.75	6.21	18.24	4.96
Total score (CABS, generalized degree of non-assertive behavior; X_9_)	16.53	10.15	27.51	9.92

**Table 4:** Results of one-factor variance analysis.

Results				
Groups	Score	Total	Medium	Dispersion
Girls’ evaluation	2	16.53	8.2646	12.6546
Boys’ evaluation	2	27.51	13.7550	40.2305

Variance analysis						
Variation source	SS	df	MS	*F*	*p*-value	*F* critical
Between the groups	30.14463	1	30.1446	1.1400	0.3975	18.5128
Within the groups	52.88505	2	26.4425			
Total	83.02968	3				

In the present study, self-esteem was conceptualized as an integral characteristic reflecting school students’ understanding of their own abilities, personal qualities, and patterns of interaction with their peers. Accordingly, the general concept of self-esteem was adopted. The operationalization of self-esteem was based on the use of the Piers–Harris Children’s Self-Concept Scale and the Children’s Assertive Behavior Scale, both of which are standardized instruments. Thus, the assessment focuses on the child’s perception of themselves, taking into account their behavior in different situations, their status at school, interactions with other students, and emotional state. The results were processed by 11 school psychologists, who considered not only the scores obtained from these scales but also the children’s behavior in the educational environment based on observational data, thereby contributing to the accuracy of the findings.

Self-esteem was operationalized at three distinct levels:

- High self-esteem was characterized by students’ confidence in their actions and active classroom engagement.
- Average self-esteem was defined by an accurate self-assessment of abilities, coupled with a fear of failure when facing challenges.
- Low self-esteem was marked by pervasive self-doubt, persistent dissatisfaction, and heightened anxiety.

Participants were categorized into passive, active-aggressive, and active-constructive communication styles based on the Children’s Assertive Behavior Scale (CABS). Furthermore, communication proficiency was classified into high, medium, and low levels based on assertive behavior patterns to ensure analytical depth:

- High proficiency was observed in students capable of maintaining dialogue, providing reasoned arguments, respecting diverse opinions, and managing conflicts effectively.
- Medium proficiency included students who could express their thoughts but lacked consistency and required external support during disputes.
- Low proficiency was identified in withdrawn students who struggled to interact independently with peers or teachers.

Quantitative data were processed using Analysis of Variance (ANOVA), Student’s *t*-tests and *χ*^2^ to identify statistically significant differences between groups.

*Phase* III: Data Interpretation. The final stage of the research design involved the interpretation of results and the validation or rejection of the research hypotheses regarding the correlation between self-esteem and communication styles.

### 3.3 Sample and data collection

Data collection was conducted using the ‘I-psychodiagnostics.kz’ SMART platform. Participants were recruited from five general secondary education institutions in the Republic of Kazakhstan. The study followed the ethical principles of voluntary participation, informed consent, and confidentiality, ensuring non-discrimination and the protection of the rights and interests of all minors involved.

The initial sample consisted of 320 middle school students (aged 14–15 years), selected via simple random sampling. Testing took place during elective psychodiagnostic sessions. Throughout the study, several participants were excluded based on predefined criteria: four students withdrew (three voluntarily and one at the request of parents), and 13 were excluded to control for confounding variables. Specifically, students presenting with acute respiratory symptoms at the time of testing were excluded to avoid bias from physical discomfort. Critically low academic achievement originally served as an exclusion criterion. No instances of such achievement emerged among the students who consented to participate and met all other inclusion criteria. The final analytical sample comprised 303 students (*N* = 303). The study was conducted during the second semester of the 2023/2024 academic year over a four-month period. Sample characteristics are detailed in Table 5.

**Table 5:** Detailed characteristics of respondents.

No	Characteristics	Frequency	Percentage
1.	Country		
	The Republic of Kazakhstan	303	100%
2.	Age		
	14 years	124	41.9%
	15 years	179	59.1%
3.	Gender/sex		
	Female	185	61.2%
	Male	118	38.8%
4.	Educational institution		
	Secondary School No. 31 (Taraz city, Zhambyl region) – urban area, public school.	71	23.5%
	Secondary School No. 47 (Taraz city, Zhambyl region) – urban area, public school.	69	22.8%
	Secondary school named after A. Moldagulova (Shushky district, Zhambyl region) – rural area, public school.	75	24.7%
	Ozat Educational Company (Taraz, Zhambyl region) – urban area, private school.	41	13.5%
	Private school ‘Al-farabi’ (Taraz city, Zhambyl region) – urban area, private school.	47	15.5%

The Piers–Harris Children’s Self-Concept Scale (Piers, 1984; Piers and Herzberg, 2002). This 80-item instrument assesses the self-concept and emotional state of children and adolescents across various age categories, evaluating their emotional status and level of social adaptation. The scale provides a personality diagnosis across six subscales that reflect different aspects of self-perception: (1) Physical Appearance and Attributes; (2) Intellectual and School Status; (3) Happiness and Satisfaction; (4) Behavioral Adjustment; (5) Freedom from Anxiety; and (6) Popularity and Social Acceptance. In this study, a 4-point Likert scale was utilized (1 = “incorrect,” 2 = “rather incorrect than correct,” 3 = “rather correct than incorrect,” 4 = “correct”). Responses were evaluated according to an interpretation key where each response is scored as 1 or 0. The total score ranges from 0 to 80, categorized as follows: High self-esteem (65–80), indicating a positive self-concept across all areas; Medium level (50–64), considered satisfactory but highlighting specific aspects requiring attention; Low self-esteem (35–49), reflecting difficulties with self-perception and confidence; and Significant difficulties (0–34), indicating profound problems across all assessed domains. For each subscale, the maximum score corresponds to the number of items included.

The Children’s Assertive Behavior Scale (CABS; Michelson and Wood, 1982; Wood et al., 1978). This questionnaire is used to diagnose communicative competence and the formation of fundamental communication skills. The scale includes 27 interpersonal scenarios grouped into five key areas: reacting to positive/negative statements, showing empathy, making requests, and initiating conversation. For each scenario, participants choose one of five responses, categorized as: “correct” (0), “incorrect passive” (−1 or −2), or “incorrect aggressive” (1 or 2). Interpretation involves summarizing the absolute values of the responses; the maximum score is 54 points. A higher score indicates a greater deviation from assertive behavior, while a lower score reflects a more confident and adaptive communication style. Additionally, the method allows for determining a propensity toward passive or aggressive styles by independently summarizing the absolute values of negative (passive) and positive (aggressive) scores, with a maximum of 27 points per subscale. This allows for an assessment of the severity and direction of non-assertive reactions in the respondent’s behavior.

### 3.4 Data analysis

Statistical and variance analyses, as well as the calculation of the Student’s *t*-test coefficient, Statistical and variance analyses (ANOVA), as well as the calculation of Student’s *t*-tests and *χ*^2^, were performed using Microsoft Excel and Statistica 12.0 software.

MS Excel was utilized for primary data processing, including the initial recording and organization of respondents’ raw scores. Advanced statistical calculations were carried out using Statistica 12.0, which enabled the generation of comprehensive tables and charts based on analytical and mathematical procedures. This automated approach to data processing ensured computational accuracy and minimized errors in determining the correlations between the studied variables.

### 3.5 Data collection

In the process of statistical analysis, the mean values (M) and standard deviation (SD) of the test results were calculated. To evaluate the statistical significance of differences between the responses of female and male students, *p*-value and F-criterion were utilized. The relationship between the variables was analyzed by gender and region using Student’s *t*-test and *χ*^2^ (Table 6).

**Table 6:** Correlation matrix of scores on the Piers–Harris Self-Concept Scale and the Children’s Assertive Behavior Scale.

Evaluation criteria	X_1_	X_2_	X_3_	X_4_	X_5_	X_6_	X_7_	X_7_	X_9_
X_1_	1								
X_2_	−0.128	1							
X_3_	0.346	0.786	1						
X_4_	−0.097	0.537	0.761	1					
X_5_	0.269	0.761	0.147	0.724	1				
X_6_	0.537	0.634	0.673	0.553	0.209	1			
X_7_	−0.673	−0.678	−0.579	−0.683	−0.237	−0.864	1		
X_8_	0.569	0.157	−0.864	−0.749	−0.491	−0.704	−1	1	
X_9_	−0.596	−0.873	−0.869	−0.834	−0.503	−0.618	−1	1	1

X_1_—assessment of physical appearance; X_2_—assessment of intellectual status; X_3_—assessment of happiness; X_4_—assessment of emotional status; X_5_—assessment of behavior; X_6_—assessment of popularity; X_7_—tendency toward passive behavior; X_8_—tendency toward aggressive behavior; X_9_—overall assertiveness deficit score. Source: compiled by the author.

### 3.6 Research ethics

The study involving middle school-aged students was reviewed and approved by the pedagogical councils of the participating educational institutions. Participation in the survey was conducted under conditions of strict anonymity. The research was carried out with the prior written informed consent of parents or legal guardians, who agreed to their child’s participation in the experiment and granted permission for the anonymized publication of the findings. Furthermore, in accordance with established ethical principles, students and their parents/guardians retained the right to withdraw from the study at any stage without penalty. Throughout the research process, the authors strictly adhered to the principles of the Declaration of Helsinki. This included a primary focus on respondents who provided voluntary informed consent and the guaranteed confidentiality of all personal and empirical data (World Medical Association, 2024).

## 4 Results

The first stage of the empirical research involved administering the Piers–Harris Children’s Self-Concept Scale to the respondents. The survey was implemented via the ‘I-psychodiagnostics.kz’ SMART platform. Within this phase of the study, the scale provided an objective assessment of the students’ self-perception across various domains of social life. The descriptive statistics and analytical results of the respondents’ scores are presented in Table 1.

The data in Table 1 indicate that self-esteem among middle school-aged children is characterized by an average (satisfactory) level. Specifically, the self-esteem scores among boys were higher (M = 56.76) than among girls (M = 54.41). These marginal differences between genders may be attributed to specific adaptive characteristics. Boys tend to exhibit greater behavioral activity and are generally less susceptible to peer influence. In contrast, girls demonstrate a higher degree of dependence on social evaluation and the perceptions of others.

However, boys may only outwardly project confidence without necessarily demonstrating active engagement in communication. Girls, conversely, tend to be more cautious and show a greater predisposition toward self-criticism. Given these observations, the practical significance of the gender-based differences in self-esteem requires further validation through inferential statistics. The results of the one-way Analysis of Variance (ANOVA) are presented in Table 2.

The results of the one-way analysis of variance did not reveal statistically significant differences in self-esteem by gender among middle-school students (*F* = 0.09; Fcrit = 4.96; *p* > 0.05). With a 95% confidence interval (*α* = 0.05), it can be concluded that gender does not impact self-esteem. Given the *p-value* (0.77) the differences in self-esteem indicators across the six subscales between female and male respondents are present but statistically non-significant. The coefficient of determination (R_2_) is 0.009, indicating that gender accounts for only 0.9% of the variance in self-esteem, while 99.1% of the variance is attributed to unaccounted factors.

The subsequent stage of the research involved examining the communication styles of middle school students using the ‘I-psychodiagnostics.kz’ SMART platform. For the 14–15-year-old age group, the Children’s Assertive Behavior Scale (CABS) was administered. The survey results facilitate an assessment of the deviation from assertive behavior toward aggressive or passive communication patterns. The descriptive and inferential statistics derived from the respondents’ scores are presented in Table 3.

Scores on the PACABS subscale indicate that a passive communication style is more characteristic of 14–15-year-old girls (M = 10.78 out of 27). Conversely, boys in this age category exhibited a more pronounced deviation on the AGCABS scale; their tendency toward an aggressive communication style was rated as above average M = 18.24 out of 27. Based on the survey results, the overall deviation from assertive behavior was 16.5 for girls and 27.51 for boys (out of a maximum 54 points). These divergent behavioral patterns appear to be rooted in gender-typed socialization. Traditionally, girls are socialized toward relational harmony, compliance, and “softness,” whereas boys are often encouraged to be agentic and assert their own interests. This is reflected in the boys’ more direct reactions to potential conflicts. In contrast, the girls’ focus on maintaining interpersonal bonds may contribute to their higher levels of communicative passivity.

To evaluate the statistical significance of gender as a factor in the formation of communication styles, a one-way Analysis of Variance (ANOVA) was conducted (Table 4):

The results of the one-way ANOVA led us to reject the hypothesis that gender exerts a statistically significant influence on the communication styles of middle school students, as the critical value Fcrit (18.51) exceeds the observed value of Ffact (1.14). At the specified significance level *α* = 0.05 and with a *p*-value is 0.39, it can be concluded that gender does not have a significant impact on communication style.

The study aimed to examine the relationship between self-esteem factors and communication style; therefore, correlation coefficients were calculated between the subscale scores of the Piers–Harris Self-Concept Scale and the Children’s Assertive Behavior Scale (CABS). The correlation matrix is presented in Table 6.

Both positive and negative associations were found between behavioral styles and evaluations of personality characteristics. Low self-esteem regarding physical appearance was moderately associated with increased communicative aggression (−0.673) and showed a positive association with passive behavior (0.569). Scores on the intellectual status scale were not related to aggression and were negatively correlated with passive communication style (−0.678). Self-esteem on the intellectual status and happiness scales showed strong negative correlations with the overall assertiveness deficit score (−0.873 and −0.869, respectively). The higher the level of life satisfaction, the lower the child’s tendency to exhibit maladaptive responses in social interactions. A similar pattern was observed for the emotional status indicator (−0.834): higher emotional stability and well-being were associated with a lower likelihood of developing aggressive or passive communication styles. Behavioral self-esteem showed no strong correlations with aggressive or passive manifestations, as well as with the overall assertiveness deficit score, indicating a limited role of this aspect in the formation of communication style among 14–15-year-old children. The null hypothesis was rejected and the alternative hypothesis was supported: a moderate to strong association exists between self-esteem and communication style in middle-school children.

A detailed assessment of the relationship between self-esteem levels and communication styles was subsequently performed, with a specific focus on gender-specific profiles. A comparative analysis of these variables was conducted using independent samples *t*-tests; the findings are summarized in Table 7.

**Table 7:** Assessment of differences in self-esteem levels and communication style among schoolchildren by gender.

Self-esteem level	Communication style	Girls		Boys		Student’s *t*-test (*t_crit_* ≈ 2,001)	*χ* ^2^
		M (% of respondents)	SD	M (% of respondents)	SD		
High	Passive	–	–	–	–	–	–
	Active-aggressive	8.1 (9%)	1.3	8.0 (6%)	1.5	0.28; *p* > 0.05	0.88; *p* ≈ 0.35
	Active-constructive	9.0 (91%)	1.8	9.2 (94%)	1.4	0.31; *p* > 0.05	0.87; *p* ≈ 0.33
Average	Passive	6.1 (43%)	1.2	6.2 (40%)	0.8	0.30; *p* > 0.05	0.84; *p* ≈ 0.27
	Active-aggressive	6.0 (18%)	0.9	6.2 (24%)	1.3	0.24; *p* > 0.05	0.29; *p* ≈ 0.33
	Active-constructive	7.2 (39%)	1.4	6.9 (36%)	1.1	0.21; *p* > 0.05	0.31; *p* ≈ 0.29
Low	Passive	4.5 (57%)	1.1	4.1 (52%)	0.8	0.39; *p* > 0.05	
	Active-aggressive	4.2 (43%)	1.3	4.7 (48%)	1.5	0.37; *p* > 0.05	0.24; *p* ≈ 0.38
	Active-constructive	–	–	–	–	–	–

Comparative analysis revealed no significant gender influence on either communicative behavior or self-esteem levels. Instead, communication styles were found to be directly contingent upon the development of self-esteem. Specifically, a passive communication style was more prevalent among both boys and girls with low self-esteem. Low self-esteem was associated with heightened insecurity and a fear of articulating personal thoughts, manifesting as social withdrawal and increased communication apprehension.

An active-aggressive communication style was observed in children with either low or significantly inflated (overestimated) self-esteem. This pattern likely reflects a defensive mechanism against external stressors, characterized by a predisposition toward conflict and a tendency to challenge collective decisions. Conversely, an active-constructive communication style was predominant among students with high and average self-esteem, enabling them to maintain effective dialogue while integrating the perspectives of others.

The self-perception of social popularity significantly determines levels of passivity and aggression through an inverse relationship: a higher subjective evaluation of social status correlates with a lower probability of maladaptive reactions during interactions with peers and the broader social environment. Consequently, these findings support the rejection of the null hypothesis in favor of the alternative: middle school students exhibit a moderate-to-strong dependence of communication style formation on self-esteem. Additional chi-square *χ*^2^ analysis showed no differences in communication styles between males and females. The results were confirmed by a *p*-value greater than 0.05, indicating that the null hypothesis was not rejected.

Given that the participants represented various educational institutions, the regional influence on the development of communication skills was also examined in relation to self-esteem levels. These findings are categorized by high, average, and low levels of communication proficiency (Table 8).

**Table 8:** Regional influence on the development of communication skills among schoolchildren.

Self-esteem level	High			Average			Low		
Communication level	High	Average	Low	High	Average	Low	High	Average	Low
Secondary School No. 31 (Taraz, Zhambyl Region), %	90	10	–	45	53	2	–	52	48
Secondary School No. 47 (Taraz, Zhambyl Region), %	95	5	–	42	57	1	–	51	49
A. Moldagulova Secondary School (Shu District, Zhambyl Region), %	92	8	–	36	62	2	–	50	50
“Ozat” Educational Company (Taraz, Zhambyl Region), %	94	6	–	35	63	2	–	53	47
“Al-Farabi” Private School (Taraz, Zhambyl Region), %	90	10	–	43	56	1	–	57	43
Overall comparison (ANOVA, *F_crit_* ≈ 2.77)	1.01	0.96	–	0.97	1.74	1.32	–	1.25	1.07
Comparison of students from rural and urban areas (*t*-Student, *t_crit_* ≈ 2.001)	0.56	0.51	–	0.38	0.41	0.25	–	0.22	0.49
Comparison of students from public and private schools (t-Student, *t*_*cri*t_ ≈ 2.001)	0.35	0.37	–	0.24	0.31	0.52	–	0.41	0.44

A comparative analysis of data between students from rural and urban cohorts revealed no statistically significant differences in self-esteem or communication proficiency. These findings underscore the critical role of self-esteem as a foundational internal resource for self-control, confidence, and assertiveness in interpersonal expression. Students with high self-esteem demonstrated a marked absence of hesitation in seeking clarification or asking questions, which facilitated their proactive engagement in communication. The lack of substantial disparities between rural and urban students, as well as between those in public and private educational institutions, can be attributed to the standardized and systematic educational framework of the Republic of Kazakhstan. This institutional consistency ensures that students, regardless of geographic location or school type, are provided with equitable opportunities to engage in the learning process and develop socio-emotional self-regulation. Consequently, self-esteem emerged as a more potent psychological predictor of communicative confidence than environmental or institutional factors. High levels of self-esteem were found to foster greater interactional flexibility and social openness across the entire sample.

## 5 Discussion

The findings suggest that at ages 14–15, students are in a critical phase of physical, psycho-emotional, and communicative maturation. The process of self-identification during middle adolescence is highly individualized, a fact supported by the high variance (standard deviation) observed in the students’ responses. This significant variability indicates that despite belonging to the same age cohort, participants exhibit diverse levels of personal development. This reinforces the premise that the experience of personal formation is unique to each student, particularly regarding the development of a communication style.

Effective social dialogue is shaped by a complex interplay of factors which, according to Smare and Elfatihi (2024), can be categorized into four domains: individual, familial, educational, and sociocultural. Among these, the authors identified openness, extraversion, and self-esteem as primary individual drivers. Our results expand upon this framework, demonstrating that self-esteem influences not only creative potential but also the core of communicative competence. In mid-adolescence, specific dimensions of self-concept such as intellectual status, perceived happiness, emotional stability, and popularity serve as the most significant predictors of how a communication style is manifested.

The empirical data confirms that a child’s self-perception directly dictates the assertiveness of their behavior. Lower evaluations of personal characteristics correlate with a higher probability of maladaptive behavioral deviations, creating preconditions for either aggression or passivity. Changes in communication styles often stem from a lack of self-acceptance and body image dissatisfaction, which is evidenced by the correlation indices in this study. Specifically, low self-evaluation of physical appearance drives deviations from assertive behavior in two directions:

- Aggression: In this scenario, the individual seeks social validation through dominance and hostility, attempting to establish status by inducing fear in peers. This correlates with the findings of Peng et al. (2022), who noted that dissatisfaction with physical appearance can trigger indirect or physical aggression fueled by underlying anger. As shown in our study, this manifests as an aggressive communication style and a significant departure from assertive norms.
- Passivity: This often serves as a defensive psychological response to bullying or cyberbullying (Prince et al., 2024). Such negative experiences inhibit the development of social skills and solidify an unattractive self-identity. More severe consequences of appearance-related dissatisfaction, including depression, anxiety, and emotional distress, are described by Merino et al. (2024). Given this aspect and the confirmed correlation between self-esteem factors and non-assertive behavior, the manifestation of unwarranted passivity in communication style is an important aspect. This indicates the necessity of psychological and pedagogical elaboration of the problem with the child in the early school age. However, this study is limited to the relationship between self-esteem levels and the behavioral characteristics of schoolchildren in social settings.

A high self-evaluation of intellectual development emerged as a cornerstone of adaptive communication. Students confident in their intellectual abilities tend to engage in dialogue based on mutual respect and emotional regulation, avoiding deviations from assertive norms. While this finding is vital for understanding adolescent behavior, it presents an interesting contrast to studies involving adult populations. In broader research, high intellectual self-evaluation is strongly correlated with behavioral plasticity and flexibility (Zajenkowski et al., 2022), which can sometimes serve as a foundation for manipulative communication styles. Consequently, the role of intellectual status in non-assertive reactions warrants further longitudinal exploration to distinguish between healthy confidence and manipulative tendencies.

Emotional development significantly shapes students’ self-esteem and their subsequent patterns of social behavior. Ofodile et al. (2025) emphasize the specific characteristics of emotional sensitivity during adolescence, suggesting that parenting styles fundamentally dictate social conduct. High family cohesion is associated with robust levels of self-esteem, while peer interaction not only enhances motivation but also facilitates social adjustment (Ofodile et al., 2025). Similarly, research by Li and Hao (2025) indicates that social support for students is instrumental in reducing anxiety and bolstering self-esteem. Such support fosters greater life satisfaction and is associated with a lower predisposition toward maladaptive assertive behavioral reactions (Li and Hao, 2025). This underscores the necessity of interpreting “happiness” as a complex, longitudinal category rather than a “short-term mental state” (Kramer et al., 2024), a perspective confirmed by our findings. While previous studies have addressed the link between emotional development and self-esteem, the connection to specific communication styles has remained relatively superficial, often overlooking gender and regional variables. Our research deepens these constructs, confirming that for 14–15-year-olds, subjective life satisfaction is a primary driver of communication style. A child who reports high levels of happiness is more likely to respect interpersonal boundaries and adhere to norms of assertiveness, maintaining a balance between personal satisfaction and communicative effectiveness.

A pivotal aspect of this study was the investigation of gender’s role in self-esteem and communication. Our results indicate that gender does not significantly influence how adolescents evaluate their own personality traits. This finding contributes to an ongoing scholarly debate; for instance, Salavera and Usán (2021) argue that gender impacts personal development, suggesting that males are more prone to active social interaction than females. Our data does not support this hypothesis, indicating no statistically significant gender effect on self-esteem or the choice of a social dialogue model among 14–15-year-olds. This aligns with the perspective of Kilby (2023), who explored the impact of gender on behavior and academic performance, noting that while communication may appear gender-related, one must account for societal stereotypes and gender expectations. While such social constructs are difficult to isolate in psychodiagnostic practice and were not the primary focus of this study, they remain a critical contextual factor. Furthermore, some scholars argue that behavior is not directly dictated by gender but is instead an effect of divergent personality types across boys and girls (Yu et al., 2021). Our research supports the notion that a child’s behavior is shaped more by an individual’s psycho-emotional structure than by biological sex. However, by providing a granular analysis of the relationship between self-esteem and communication styles, our findings did reveal specific gender-based distributions in behavioral manifestations. The findings thus align with the tenets of sociometer theory, which posits that self-esteem reflects an individual’s subjective perception of their acceptance and significance within the social environment. The identified associations between self-esteem levels and communication styles indicate that higher self-esteem relates to constructive peer interactions, whereas less favorable self-esteem correlates with less effective communicative strategies. These data comprehensively support the conceptualization of self-esteem as a vital psychological mechanism linked to the success of adolescent social interaction. The existing inconsistencies in international findings suggest the presence of unaccounted mediating factors, highlighting the need for more in-depth, longitudinal studies.

## 6 Conclusion

The findings of this study demonstrate robust correlations between specific self-esteem indicators and the behavioral patterns of adolescents aged 14–15. During this critical developmental stage, intense psychological and social transformations often lead to fluctuations in interpersonal communication styles, frequently resulting in deviations from assertive behavior toward either passivity or aggression.

The assessment of self-perception revealed only marginal gender-based differences. While male participants exhibited a slightly higher overall self-evaluation potentially due to a lower susceptibility to peer influence, they also demonstrated more prominent instances of non-assertive behavior. Specifically, a predisposition toward aggressive communication was found to be context-dependent, manifesting primarily during interpersonal conflicts.

A definitive relationship was established between self-esteem levels and communicative modalities:

- High self-esteem serves as a catalyst for an active-constructive communication style, regardless of gender.
- Conversely, low or inflated (overestimated) self-esteem is consistently associated with maladaptive patterns, including passive withdrawal or active-aggressive reactions.

Furthermore, the data indicate that regional and institutional factors do not significantly impact the development of communication skills in the studied cohort. Instead, these skills are primarily driven by internal self-regulatory mechanisms. Consequently, psychological interventions aimed at bolstering self-esteem are likely to yield a significant reduction in non-constructive interactions while promoting proactive social engagement.

The results of this research offer a valuable empirical foundation for enhancing educational and counseling practices. Specifically, the findings can be utilized to:

1. Develop targeted confidence-building programs and anxiety-reduction protocols for middle school students.
2. Design communication skills training tailored to address specific self-esteem profiles.
3. Inform the creation of socio-emotional learning (SEL) curricula that prioritize internal psychological resources over external environmental factors.

### 6.1 Limitations

This study has several important limitations. First, the research was conducted with middle school-aged children (14–15 years old) who attend public schools in Kazakhstan and were not identified by psychologists or teachers as having significant behavioral issues. Therefore, the findings cannot be generalized to the broader population of adolescents of this age group, as multiple contextual and individual factors, which can influence on their behavior—including the classroom climate, peer relationships, parent–child interactions, and broader socio-cultural dynamics.

Furthermore, this age group is characterized by specific developmental features, including unique trajectories in personal development, communication skills acquisition, and level of socialization. These individual differences must be taken into account when interpreting psychological assessments and forming conclusions. Another significant limitation is the correlation nature of the analysis. While the study identifies associations between variables, it does not allow for causal inferences or precise conclusions regarding direct or indirect effects. Longitudinal or experimental designs would be required to explore causality more thoroughly.

The study sample relies on a convenience selection of public school students from a single region of Kazakhstan, restricting the generalizability of the findings to adolescents from other regions of the country or alternative cultural and educational contexts. Regional, sociocultural, and institutional characteristics potentially shape the development of self-esteem and communication styles, which required interpreting the findings strictly within the context of the investigated sample. Testing the robustness of the identified patterns necessitates future research involving more diverse and representative samples.

### 6.2 Perspectives for further research

Analysis of modern traditional and innovative (using ICT) methods of psychodiagnostics counseling of school-age children in order to correct the communication style of pupils and eliminate non-assertive reactions in their behavior. Comparison of the techniques’ effectiveness by experimental way and introduction into psychodiagnostics practice in schools of the Republic of Kazakhstan. The most effective psychotherapeutic practices will be adapted to the format of SMART-platform ‘I-psychodiagnostics.kz’ and will complement its functionality. In further research, we plan to focus on the intercultural aspect, which will involve students from other countries, not just different schools in Kazakhstan.

## Data Availability

The original contributions presented in the study are included in the article/supplementary material, further inquiries can be directed to the corresponding author.
